# The role of lipophagy in liver cancer: mechanisms and targeted therapeutic interventions

**DOI:** 10.3389/fcell.2025.1562542

**Published:** 2025-07-08

**Authors:** Peiyu Han, Han Wang, Yuyu Chen, Yuqiu Ge, Huiting Xu, Hongbo Ren, Yiteng Meng

**Affiliations:** ^1^ Department of Gastroenterology, Shenzhen People’s Hospital (The Second Clinical Medical College, Jinan University, The First Affiliated Hospital, Southern University of Science and Technology), Shenzhen, Guangdong, China; ^2^ Wuxi School of Medicine, Jiangnan University, Wuxi, Jiangsu, China; ^3^ Clinical Laboratory Affiliated Tumor Hospital of Nantong University & Nantong Tumor Hospital, Nantong, China; ^4^ Department of Gastroenterology, Qilu Hospital, Jinan, Shandong, China

**Keywords:** lipophagy, liver cancer, cancer progression, inhibition and promotion of cancer, targeted medicine

## Abstract

Liver cancer, the advanced stage of various chronic liver diseases, has garnered attention due to its high incidence and insidious progression. Lipid droplets (LDs), unique lipid storage organelles in hepatocytes, play a pivotal role in lipid metabolism. Lipophagy, a selective autophagy process initially identified in hepatocytes, regulates lipid homeostasis by selectively degrading LDs. This process offers a novel therapeutic avenue for addressing lipid metabolism disorders in liver cancer. This review highlights the regulatory role of lipophagy in liver cancer progression and its therapeutic potential. It elaborates on the molecular mechanisms underlying lipophagy-mediated LDs degradation and discusses the dual regulatory role of lipophagy in liver cancer. While lipophagy can suppress liver cancer development, under specific conditions, it may promote cancer cell proliferation, inhibit apoptosis, facilitate invasion and metastasis, and contribute to treatment resistance. Consequently, strategies targeting lipophagy for liver cancer prevention and therapy hold significant promise. These include interventions through traditional Chinese and Western medicine, as well as lifestyle modifications. This review evaluates current research, hotspots, and controversies in the field, aiming to provide innovative therapeutic strategies for liver cancer associated with abnormal lipid metabolism.

## 1 Introduction

Autophagy, an adaptive mechanism, is activated in response to extreme intracellular and extracellular stress, maintaining cellular functions and defending against pathogen invasion (White et al., 2021). Under starvation, it non-selectively degrades cytoplasm, while in nutrient-rich conditions, it selectively removes substances via lysosomes (Zhang and Ghaemmaghami, 2016). LDs, eukaryotic organelles that store lipids, play a crucial role in cellular energy management (Haidar et al., 2021). During starvation, cells hydrolyze triglycerides (TGs) in LDs to release free fatty acids (FFAs) for beta-oxidation. The transport of intracellular lipids to lysosomes via autophagosomes for decomposition is termed lipophagy (Shi et al., 2019). First defined in 2009, lipophagy mediates lysosomal lipid transport via autophagosomes (Singh et al., 2009) and has been shown to degrade LDs through the autophagy-lysosome pathway in various organ systems (Zhou et al., 2019).

The liver, as the central organ for fat synthesis and lipid oxidation, is integral to lipid metabolism (Bechmann et al., 2012). In the liver, lipophagy plays critical physiological and pathological roles, with impaired hepatic lipid metabolism leading to abnormal fat accumulation. Nonalcoholic Fatty Liver Disease (NAFLD), characterized by a spectrum ranging from simple steatosis to cirrhosis and liver cancer, exemplifies hepatic metabolic dysfunction (Friedman et al., 2018). Such dysfunction is closely associated with liver diseases, including hepatitis, cirrhosis, and liver cancer.

Liver cancer, a prevalent malignant tumor, involves complex pathogenesis regulated by numerous signaling pathways and molecular mechanisms (Wang and Deng, 2023). Metabolic syndrome is an independent risk factor for hepatocellular carcinoma (HCC) (Fu et al., 2023). In the tumor microenvironment, where oxygen and nutrients are scarce, tumor cells adapt by altering lipid metabolism. Enhanced lipid acquisition, production, and storage are hallmarks of invasive tumors, with beta-oxidation providing critical energy for tumor survival (Behne and Copur, 2012; Kounakis et al., 2019). Accordingly, drugs targeting lipophagy in liver cancer cells have emerged as promising tools for reducing liver cancer risk and improving therapeutic outcomes.

## 2 Molecular mechanisms of lipophagy

Lipophagy, crucial for intracellular lipid metabolism, involves multiple molecular components and offers targets for liver cancer treatment. It maintains lipid balance under normal conditions but degrades LDs in stressful environments. Abnormal lipophagy can promote liver cancer. Thus, investigating its mechanisms can reveal roles and new therapeutic targets.

### 2.1 Lipophagy targeting lipid droplet degradation

Currently, the known forms of autophagy include macroautophagy, microautophagy, and chaperone-mediated autophagy (CMA). The autophagy of LDs may occur through any of these pathways (Li et al., 2024), confirming its role as selective autophagy. Similar to a wide range of selective autophagy processes, lipophagy has three main routes: macrolipophagy, microlipophagy, and chaperone-mediated autophagy. The specific form that occurs depends on how the LDs are transported to the lysosome.

In macrolipophagy, AMP-activated protein kinase (AMPK) induces macrolipophagy by activating UNC-51-like kinase 1 (ULK1). At the transcriptional level, the transcription factor (TF) EB promotes lysosomal biogenesis and macroautophagy (Han et al., 2023). CMA serves as a critical upstream regulator for both macrolipophagy and cytoplasmic lipolysis. Sirtuin 3 (SIRT3) activates AMPK, thereby enhancing macroautophagy and CMA, which aids in reducing the accumulation of LDs within hepatocytes. Heat Shock Cognate Protein 70 (Hsc70) binds to Perilipin (PLIN) 2, while AMPK phosphorylates PLIN2, triggering its post-translational modification. At the transcriptional level, the nuclearrespiratoty factor 2 (NRF2) promotes the expression of the Lysosomal-Associated Membrane Protein (LAMP) 2A gene, thereby facilitating CMA activity (Kaushik and Cuervo, 2015; Kaushik and Cuervo, 2016). Microlipophagy directly attracts LDs to lysosomes for phagocytosis and degradation (Yu and Li, 2017).

### 2.2 Regulatory roles of lipophagy-related factors

Lipophagy is influenced by genes, transcription factors, enzymes, etc.

#### 2.2.1 Lysosome-associated membrane proteins

Autolysosome formation during lipophagy depends on various factors. Studies have shown that LAMP2 deletion in mouse fibroblasts enlarges autolysosomes, enhances lipophagy, and increases LD degradation via the ceramide analog N-(1-hydroxy-3-morpholinopropyl-2-yl) decanamide (Kato et al., 2020). The interaction between phosphatidylethanolamine (PE), autophagy-related gene (Atg) 14, ULK1, and microtubule-associated protein 1 light chain 3 (LC3) induces lipophagy, releasing free fatty acids.

Lee et al. reported that serum absence and oleic acid (OA) inhibit LC3-LAMP1 colocalization, preventing autolysosome formation. Upon OA removal, lipid accumulation decreases, and autophagic flux recovers due to starvation-induced lipophagy (Lee et al., 2019). Cui et al. suggested that fatty acids from lipophagy do not directly cross lysosomal membranes but require lysosome-plasma membrane fusion, followed by exocytosis. This process is regulated by the lysosomal calcium channel protein mucolipin 1 (Cui et al., 2021).

#### 2.2.2 ATGL and its family lipases

Adipose Triacylglyceride Lipase (ATGL) and Patatin Like Phospholipase Domain Containing (PNPLA) 8 act as selective autophagy receptors in lipophagy (Sathyanarayan et al., 2017; Kim et al., 2011). ATGL and homologous lipases, including PNPLA1, PNPLA3, PNPLA5 and PNPLA8, initiate lipophagy by facilitating autophagosome formation from triglycerides and cholesteryl esters (CE). ATGL, essential for lipolysis, plays a key role in lipophagy by interacting with the LC3 interaction region domain, enabling cytoplasmic ATGL to move toward LDs and induce lipophagy (Sathyanarayan et al., 2017). ATGL also promotes lipophagy by regulating intracellular LD degradation in the liver through SIRT1 activity.

Lipases associated with LDs, such as PNPLA5, contribute to lipophagy and autophagic proteolysis (Dupont et al., 2014). These lipases recruit triglycerides and sterol esters to initiate lipid phagocytosis and autophagosome formation (Morel and Codogno, 2018). PNPLA8 mediates Sterol Regulatory Element Binding Protein 3 (SREBP 3) driven lipophagy via interaction with LC3 in hepatocytes of mice on high-fat diets (HFD). PNPLA3 is crucial for autophagosome formation during lipid phagocytosis in starved human hepatocytes (Negoita et al., 2019).

#### 2.2.3 Rab GTPases

Rab GTPase-activating proteins (Rab GAPs) regulate vesicle trafficking and serve as markers for organelles and vesicles within the endocytosis and secretion systems. Rab family members, such as Rab7, Rab10, and Rab15, are critical regulators of lipophagy and lipophagy in the liver. Recent studies identified Rab7 and Rab10 as molecular switches promoting lipid phagocytosis in hepatocytes (Li et al., 2020). Loss of Rab7 or Rab10 impairs lipid phagocytosis, leading to LD accumulation.

Rab7 is activated under nutrient deprivation and facilitates the recruitment of multivesicular bodies and lysosomes to LD surfaces, reducing hepatocyte lipophagy capacity (Xing et al., 2021). Rab10 forms a complex with EH domain-binding protein 1 and EH domain-containing protein 2, promoting LC3-positive autophagic membrane migration to LD surfaces (Li et al., 2016). Rab10 loss results in LD accumulation. Lipophagy regulation depends on nutritional status and involves receptors and proteins, including the Farnesoid X receptor, Peroxisome proliferator-activated receptor α (PPARα), cAMP-response element binding protein, mammalian target of rapamycin (mTOR), and AMPK.

#### 2.2.4 Transcription factor

Transcriptional and post-transcriptional regulation of lipophagy involves factors such as TFEB, TFE3, Forkhead box class O proteins (FOXOs), and glycine N-methyltransferase. Under nutrient deprivation, TFEB regulates lysosomal lipase expression in mouse hepatocytes. Xiong et al. demonstrated that TFEB overexpression in hepatocellular carcinoma cells enhances LC3-LD association and reduces intracellular lipid content (Xiong et al., 2016). TFEB binds to key promoters during lysosomal biogenesis, modulating transcription (Martina et al., 2014). Conversely, TFEB knockout leads to LD accumulation in hepatocytes (Settembre et al., 2013). TFE3 induces lipophagy in hepatocytes, while Forkhead box protein O (FOXO) 1 associates with lysosomal lipases and triggers lipophagy in adipocytes during fasting (Lettieri Barbato et al., 2013). In a mouse model of Dalton’s lymphoma, Patra et al. showed that gamma radiation inhibits lipophagy by altering electrochemical properties via NRF2, enhancing the anticancer effect of gallic acid through superoxide dismutase-mediated apoptosis (White, 2015).

Therefore, lipophagy regulation is a fine network of complex molecules and processes, and interactions between core molecules and signaling pathways are key, which respond to environmental changes, maintain cellular homeostasis, and safeguard normal life activities (as shown in Supplementary Tables S1–S3).

## 3 Regulatory role of lipophagy in the progression of liver cancer

Lipophagy exhibits stage-dependent duality in HCC, inhibiting tumor growth in early stages through lysosomal degradation of cytotoxic lipids while fueling metastatic progression in advanced disease via FFA β-oxidation within hypoxic niches (Gómez de Cedrón and Ramírez de Molina, 2016).

### 3.1 Inhibition of liver cancer development by lipophagy

#### 3.1.1 Lipid breakdown is a key step in the anticancer effect of lipophagy

Excessive LD accumulation is linked to liver diseases and disorders, leading to chronic injury, fibrosis, cirrhosis, and cancer (Berardi et al., 2022; Liu K. et al., 2022; Chen et al., 2023; Zhou et al., 2018). Reducing LDs is critical. Lipophagy shows promise in early liver cancer by degrading LDs, reducing stress and inflammation, and preventing DNA damage. Chi et al. found suppressing Atg7 increased LD accumulation, stress, and DNA damage, activating p53 and promoting liver cancer in a model (Chi et al., 2016). Similarly, Xue et al. observed that inhibiting Atg5 or Atg7 in a diethylnitrosamine (DEN)/HFD-induced liver cancer model elevated hepatocyte LDs and inflammatory cytokines (IL-6, TNF-α, IL-1β), activating nuclear factor kappa-B (NF-κB) and STAT3 pathways, which facilitated liver cancer progression (Xue et al., 2016).

Beyond tumor suppression, lipid degradation provides energy substrates and intermediates for rapidly proliferating cancer cells, supporting their survival (Snaebjornsson et al., 2020). These findings suggest that lipophagy is an effective tumor-suppressive mechanism.

#### 3.1.2 Lysosomal acid lipase as another participant in lipophagy’s anti-cancer role

Lysosomal Acid Lipase (LAL) is a key lipase promoting lipid phagocytosis with tumor-suppressive activity. Zhao et al. found insufficient LAL expression accelerates tumor growth and metastasis by activating myeloid-derived suppressor cells (MDSCs) (Zhao et al., 2015). LAL functions in acidic pH, hydrolyzing CE and TG in LDs to release free fatty acids for energy (Martinez-Lopez et al., 2016; Li and Zhang, 2019).

Du et al. found hepatocyte-specific LAL expression in a mouse model suppressed melanoma metastasis (Du et al., 2015). Furthermore, research by Ouimet et al. revealed LAL’s dual role in CE metabolism, participating in CE hydrolysis both in the endoplasmic reticulum and within LDs (Ouimet et al., 2011). LAL activity is closely associated with tumor progression and metastasis, underscoring its potential as a tumor suppressor (Qu et al., 2009; Wang et al., 2023). LAL deficiency causes hematopoietic abnormalities, increasing MDSCs that suppress immune surveillance and stimulate tumor development (Zhao et al., 2016). Studies have also indicated that LAL expression improves lipid metabolism and reduces lung and liver cancer metastasis (Wang et al., 2023).

Although these findings remain preliminary, they offer valuable insights into the underappreciated role of lipophagy in cancer metabolism. This evidence highlights the need for further research to better understand the role of lipophagy in cancer, which may vary depending on tumor type and stage.

#### 3.1.3 Regulation of other signaling pathways by lipophagy to prevent the progression of liver cancer

The progression of liver cancer is driven by dysregulated signaling pathways and genetic alterations triggered by inflammatory responses, which lead to tumor formation. Inhibiting inflammation may prevent or slow HCC progression.

In liver cancer cells, siRNA-mediated Rab7 downregulation leads to abnormal LD accumulation. Conversely, in nutrient-deprived hepatocytes, Rab7 is recruited to LD surfaces, activating targeted degradation via lipophagy (Bucci et al., 2000; Jäger et al., 2004; Schroeder et al., 2015). Furthermore, LAL, which participates in lipophagy, exhibits anticancer properties. Defects in LAL stimulate tumor growth through mTOR pathway regulation and activation of MDSCs, enabling immune evasion of metastatic tumor cells (Zhao et al., 2016; Ding et al., 2014). Abnormal activation of mTOR in liver cancer can lead to chemotherapy resistance, and there exist two complexes, mTORC1 and mTORC2. Lipophagy can inhibit mTOR activity through negative feedback, enhancing chemotherapy sensitivity. For instance, inducing lipophagy with OA in HepG2 cells reduces mTORC1/2 activity and increases cisplatin sensitivity (Feng et al., 2019). Similarly, OA-induced lipophagy in Huh7 cells decreases mTORC1/2 activity and enhances docetaxel sensitivity (Song et al., 2021). These findings suggest that lipophagy improves chemotherapy efficacy in liver cancer by suppressing mTOR signaling.

In liver cancer, the inhibition of AMPK signaling reduces autophagy, promoting tumorigenesis (Sharma et al., 2023; Wang L. et al., 2019; Sun et al., 2019). Lipophagy activates AMPK through positive feedback, increasing autophagy levels and suppressing tumor progression. For example, Song et al. found that metformin induced autophagy through a PRKA-independent pathway in a diabetes-mimicking ob/ob mouse model, increasing autophagosome and autolysosome formation in hepatocytes (Song et al., 2015; Lai et al., 2020). Metformin may therefore enhance autophagy and suppress liver cancer development in cellular and animal models.

Mutations or activation of the p53 pathway in liver cancer can reduce cell apoptosis and senescence, thereby promoting tumor growth (Zhu et al., 2023; Liang et al., 2022). Lipophagy activates p53 signaling via positive feedback, increasing apoptosis and inhibiting tumor progression. Studies by El-Fakharany et al. and Wang et al. demonstrated that OA-induced lipophagy increased p53 expression and activity, suppressing liver cancer development (El-Fakharany et al., 2023; Wang et al., 2021).

NF-κB promotes inflammation and proliferation, and its activation in liver cancer fosters a tumor-promoting microenvironment (Chen L. et al., 2022; Vucur et al., 2023). Lipophagy can inhibit NF-κB signaling via negative feedback, reducing inflammatory factor expression and tumor development. For example, Wu et al. found that OA-induced lipophagy in a liver cancer mouse model reduced NF-κB nuclear translocation and the expression of IL-6, TNF-α, and MCP-1, suppressing tumor occurrence (Wu CC. et al., 2022).

STAT3 enhances the proliferation and invasion of liver cancer cells (Makino et al., 2023). Lipophagy can suppress STAT3 signaling through negative feedback, reducing the expression of growth and invasion-related factors. Ishteyaque et al. demonstrated that OA-induced lipophagy in a DEN-induced hepatocellular carcinoma model decreased STAT3 phosphorylation and the expression of vascular endothelial growth factor and Matrix metalloproteinase 9, thereby inhibiting liver cancer progression (Ishteyaque et al., 2024).

### 3.2 Lipophagy promotes the occurrence and development of liver cancer

#### 3.2.1 Lipophagy participates in the energy metabolism of tumor cells by releasing free fatty acids

Although lipophagy inhibits liver cancer initiation, evidence suggests it also promotes tumor progression (Tian et al., 2019; Patra et al., 2020). In the tumor microenvironment, characterized by inadequate blood vessel formation, nutrient scarcity, and hypoxia, tumor cells face severe metabolic stress. To adapt, they activate metabolic pathways to obtain energy and biosynthetic materials, supporting rapid proliferation and invasion. Lipophagy plays a central role by releasing FFAs to meet these demands (Kim et al., 2020). FFAs regulate signaling pathways that affect tumor cell proliferation, apoptosis, autophagy, and invasion while maintaining lipid homeostasis and energy balance (Chung et al., 2023).

Lei et al. found that inhibiting autophagy by targeting autophagy-related genes Atg5 or Atg7 with siRNA in HepG2 cells reduced FFAs, impaired mitochondrial function, increased ROS levels, induced cell cycle arrest, and promoted apoptosis (Lei et al., 2021). Similarly, Chen et al. reported that suppressing autophagy in Huh7 cells via siRNA targeting Beclin1 or Atg7 reduced FFAs, impaired mitochondrial function, increased lactate levels, and diminished cell migration and invasion (Chen LJ. et al., 2022). These findings indicate that lipophagy can promote liver cancer progression by supporting tumor metabolism.

#### 3.2.2 Lipophagy promotes liver cancer cell proliferation and inhibits liver cancer cell apoptosis

In liver cancer, the activation of the Hypoxia-Inducible Factor 1α (HIF-1α) pathway often promotes tumor progression. Lipophagy positively feeds back to activate HIF-1α and upregulates adaptive genes. Zhu et al. showed that overexpression of Beclin1/Atg7 induces lipophagy, stabilizes HIF-1α, upregulates target genes, and enhances tumor proliferation and invasion (Zhu et al., 2022).

Denk et al. reported that the autophagy-related protein p62, a selective autophagy substrate, mediates interactions in various signaling processes. While p62-related pathways can prevent genomic damage and carcinogenesis, they also support tumor cell survival and progression (Denk et al., 2019).

Cluster of differentiation 36 (CD36)-mediated uptake of oxidized low-density lipoprotein in hepatocytes activates CCAAT/enhancer-binding protein β (C/EBPβ), increasing endoplasmic reticulum proteins and promoting lipophagy, which accelerates liver cancer progression (Tian et al., 2019). Mukhopadhyay et al. found that Atg14 overexpression in HeLa cells inhibited viability, increased mitochondrial apoptosis, and triggered endoplasmic reticulum stress. However, inhibiting Atg14-induced lipophagy enhanced apoptosis, suggesting a role for lipophagy in tumor cell survival (Mukhopadhyay et al., 2017).

These findings highlight increased lipophagy levels in liver cancer cells, allowing them to exploit lipid resources for rapid growth. By activating lipophagy, tumor cells sustain their metabolic needs and enhance survival mechanisms.

#### 3.2.3 Lipophagy involves in the invasion and metastasis of liver cancer cells and affects the drug resistance of liver cancer treatment

Clement et al. showed that in obesity, cancer cells LD lipophagy release fatty acids for oxidation and increase EVs for oxidation and invasion (Clement et al., 2020). Similarly, Wang et al. showed that cancer-associated fibroblasts (CAFs) were metabolically active, with increased lipid content and lipophagic activity. CD36 or regulated CAFs, via reprogramming of tumor cell lipid metabolism, shadowed tumor proliferation and migration (Wang et al., 2024). Research indicates that lipophagy plays a critical role in drug resistance in liver cancer treatment. Sun et al. found that inhibiting miR-425 promotes lipophagy via autophagic processes, contributing to sorafenib resistance. By combining lipophagy activation with standard chemotherapy, LD levels can be reduced, enhancing chemotherapy efficacy. Thus, targeting lipophagy-mediated LD degradation represents a novel strategy for overcoming drug resistance in liver cancer (Sun et al., 2021). Lipophagy has a dual role in drug resistance. It can either promote or counteract the development of drug resistance, and this dual role depends on the specific substance type and cellular environment (as shown in Supplementary Table S2).

## 4 Potential liver cancer prevention and treatment strategies by targeting lipophagy

HCC development is a multistage carcinogenic process involving dysregulated signaling pathways, inflammatory response activation, and genetic alterations (Scaggiante et al., 2014). Early and precise interventions targeting lipophagy are crucial to improving treatment success rates.

### 4.1 Lipid droplet degradation induced by lipophagy is an important aspect of liver cancer treatment

In liver cancer, LD accumulation is closely associated with tumorigenesis and aggressiveness (Berardi et al., 2022; Scorletti and Carr, 2022). Zhang et al. highlighted the cancer-protective effects of lipophagy, including lipid breakdown to provide energy for cellular functions, prevention of ATP depletion that causes mitochondrial dysfunction, and metabolism of potentially toxic lipid molecules (Zhang et al., 2018). Schroeder et al. found that nutrient deprivation directly activates Rab7 on LD surfaces, promoting the recruitment of degradative structures and triggering targeted LD degradation via lipophagy (Schroeder et al., 2015). Wu et al. showed HCC cells inhibit FAO in response to sorafenib, leading to LD accumulation. Undegraded fatty acids convert to LDs via AKR1C3, reducing lipotoxicity and ROS, promoting HCC cell survival (Wu C. et al., 2022). Thus, inhibiting lipophagy may emerge as a potential therapeutic strategy for HCC. Understanding the role of lipophagy in LD degradation is crucial for elucidating liver cancer’s metabolic mechanisms and developing novel treatments.

### 4.2 Regulatory role and therapeutic prospects of key factors in lipophagy in liver cancer

Numerous studies have shown that lipophagy is upregulated in liver cancer, with factors like C/EBPα (Lu et al., 2015), PTPRO (Zhang et al., 2015), and p53 (Tian et al., 2015) playing pivotal roles. In mouse models, LAL deficiency significantly inhibits the progression of liver B16 malignant tumors (Du et al., 2015), highlighting lipophagy’s importance and LAL as a potential therapeutic target. Strategies targeting these factors to regulate lipophagy hold promise for HCC therapy. Future research may uncover additional mechanisms through which lipophagy influences cell physiology, making it a viable therapeutic target.

Mukhopadhyay et al. reported Atg14-induced lipophagy causes FFA accumulation and ER stress-mediated apoptosis. Inhibiting lipophagy in HeLa-Atg14 cells improves viability, suggesting new liver cancer treatment strategies (Mukhopadhyay et al., 2017). Schroeder et al. found Rab7, enriched in LDs under nutrient deficiency, promotes degradative structure recruitment, exhibits antiproliferative properties, and reduces tumor aggressiveness, positioning it as a potential therapeutic target for hepatic lipophagy (Schroeder et al., 2015). MicroRNAs (miRNAs) also play critical roles in the diagnosis and treatment of diseases. MiR-30b-5p regulates lysosomal biogenesis and autophagy by inhibiting TFEB-dependent transactivation (Guo et al., 2021), while miR-155 modulates alcohol-induced autophagy via the mTOR pathway and lysosomal proteins LAMP1 and LAMP2 (Babuta et al., 2019). Inhibition of miR-214-3p upregulates Ulk1 expression, enhancing autophagy and reducing fatty liver disease severity (Lee et al., 2021). Given that fatty liver disease can progress to hepatocellular carcinoma, miRNAs may represent therapeutic targets for liver cancer treatment by modulating autophagy or lipophagy.

Investigating the molecular mechanisms of lipophagy’s core regulatory factors provides a new perspective for liver cancer treatment. Deeper understanding of these factors may lead to more effective therapies and improved patient outcomes. Future research should focus on precise regulation of lipophagy and translating these findings into innovative clinical applications.

### 4.3 Research progress on the prevention and treatment of liver cancer through targeting lipophagy with traditional Chinese and Western medicines

Hepatocytes can regulate the initiation and progression of lipophagy under various stresses by relying on specific transcription factors and signaling pathways. In the field of exploring the prevention and treatment of liver cancer, traditional Chinese medicines (TCM) and western medicine have been found to precisely regulate liver lipid metabolism by affecting lipophagy. Therefore, a deep understanding of these mechanisms opens up new directions for the development of effective prevention and treatment strategies for liver cancer.

TCM components have been shown to regulate lipophagy, influencing liver cancer progression. For instance, quercetin, an antioxidant, promotes lipophagy by reducing PLIN2 levels, inducing AMPK activity, and enhancing LC3-II-PLIN2 colocalization in the liver (Zeng et al., 2019). Zhou et al. found that phillyrin restores lysosomal biogenesis and lipid phagocytosis through TFEB, reduces inflammation, and accelerates lipid clearance via Ca^2+^ induction (Zhou et al., 2022). Nobiletin alleviates hepatic steatosis by mediating lysosomal biogenesis and lipophagy (Yang X. et al., 2022). Similarly, leonurine and Cyclocarya paliurus extracts promote hepatic lipid clearance through the lipophagy pathway (Zhang et al., 2021). Formononetin enhances hepatic steatosis in mice by activating AMPK, promoting TFEB nuclear translocation, increasing lysosomal biogenesis, unblocking autophagic flux, and inducing lipophagy (Wang Y. et al., 2019). These TCM components reduce lipid accumulation and tumor growth in liver cancer cells by activating specific signaling pathways.

Western medicines such as dimeric procyanidins and cannabidiol, autophagy activators, promote LD degradation, and improve fat storage, making them potential therapeutic targets for liver diseases (Han et al., 2023). Park et al. reported that metformin promotes lipophagy by acting on the AMPK and AMPK-SIRT1 axis, alleviatiing excessive fat accumulation, reducing necroptosis, and mitigating hepatic steatosis (Park et al., 2023). Jung et al. found that a p62 agonist promotes lipophagy through the N-degron pathway, with therapeutic benefits observed in mouse models of fatty liver and obesity (Jung et al., 2023). Zhou et al. showed that CAY10566, a specific SCD1 inhibitor, enhances AMPK activity, promoting lipophagy and significantly reducing hepatic steatosis and LD accumulation (Zhou et al., 2020). Ma et al. revealed that resveratrol reduces oxidative stress by downregulating HIF-1α protein expression and mitochondrial ROS production in the liver (Ma et al., 2017). Marina et al. found that mTORC1 can stimulate this pathway to augment lipophagy, shielding the liver from lipid toxicity and presenting treatment strategies for liver cancer (Garcia-Macia et al., 2021).

**FIGURE 1:**
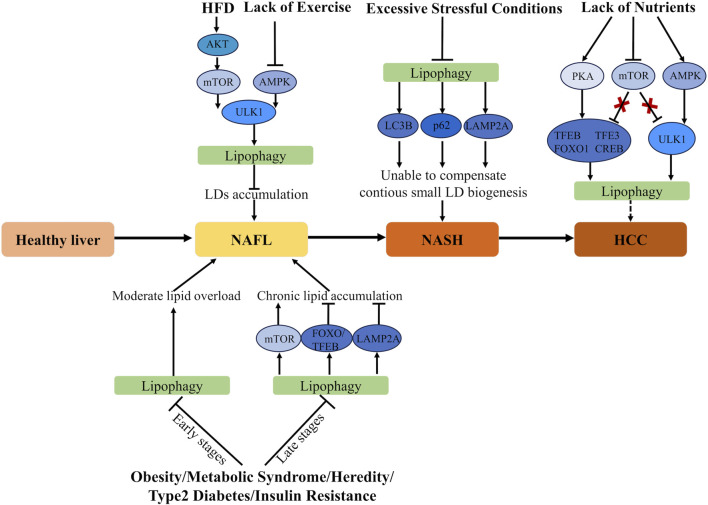
Schematic diagram of the effects of drug metabolism and signal regulation on hepatocyte lipophagy. PPARα, Peroxisome proliferator-activated receptor α; Sirt1, sirtuin-1; FFA, free fatty acid; ROS, Reactive oxygen species; IR, Insulin resistance; LKB, Liver kinase B.

Early-stage HCC may respond to pro-lipophagy agents like resveratrol, which promote lipid clearance and increase chemosensitivity. In contrast, advanced or metastatic HCC relies on lipophagy for energy production, making inhibitors such as chloroquine potential candidates for inducing tumor nutrient deprivation. Given the heterogeneity of HCC, tailored therapeutic strategies are essential. Clinical implementation should integrate precision medicine approaches with multimodal therapies to maximize treatment outcomes. Both traditional Chinese and Western pharmaceuticals could target lipophagy pathways, mitigating hepatic steatosis by reducing lipid accumulation and serving as potential interventions for HCC (as shown in Supplementary Table S3; Figure 1).

### 4.4 Research progress on the prevention and treatment of liver cancer through lifestyle regulation by lipophagy

For liver cancer patients, moderate exercise, a balanced diet, and limiting high-fat and high-sugar food intake are effective management strategies. Wu et al. showed exercise and diet adjustments reduce hepatic triglyceride accumulation via AMPK/ULK1 activation and AKT/mTOR/ULK1 inhibition, enhancing lipophagy. Exercise boosts FGF21 release, driving hepatic lipophagy through AMPK (Gao et al., 2020). These findings suggest that adhering to a healthy lifestyle with regular eating habits and moderate exercise may help prevent liver cancer.

Excessive carbohydrate intake can lead to intestinal lipid accumulation, but the ROS-AKT-Beclin1 pathway stimulates lipophagy in intestinal cells to reduce this buildup (Wu LX. et al., 2022). Additionally, High-phosphorus diets also promote lipophagy via AMPK and reduce liver lipid deposition (Liu X. et al., 2022). Fasting triggers FGF21 signaling to activate lipophagy and lipid catabolism. Refeeding after starvation stimulates intestinal lipophagy (Byun et al., 2020). Raimundo et al. demonstrated that reintroducing food after starvation stimulates intestinal lipophagy activity (Raimundo, 2022). While nutritional deficiencies do not directly cause liver cancer, they increase risk by altering metabolic and lipophagic processes regulated by PKA, mTOR, and AMPK during starvation (Shi et al., 2019; Komatsu et al., 2005).

Recent studies show that exercise induces lipophagy, influencing LD dynamics and promoting lipid metabolism in hepatocytes (Gao et al., 2020; Pino-de la Fuente et al., 2022; Li et al., 2021). For instance, 15 weeks of treadmill exercise alleviated hepatic steatosis, inflammation, and liver damage caused by a high-fat diet (HFD) in mice (Yang Y. et al., 2022). Another study reported that 8 weeks of exercise reduced liver damage and LD size in hepatocytes of mice with fatty liver disease. Exercise also enhances FGF21 production, which promotes lipophagy via AMPK in the liver (la Fuente et al., 2019).

**FIGURE 2:**
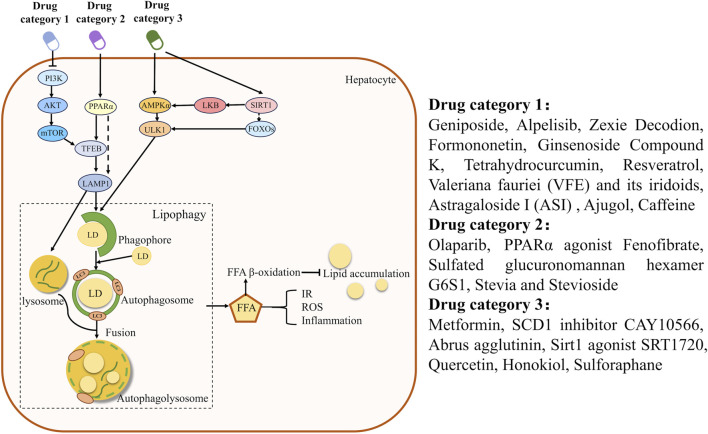
The role of lipid metabolism network and lipophagy in the spectrum of liver cancer diseases. PI3K, Phosphatidylinositol-3-kinase; mTOR, Mammalian target of rapamycin; AMPK, AMP-activated protein kinase; TFEB, transcriptional factor EB; FOXO, Forkhead box O; LAMP, Lysosomal-associated membrane protein; AKT, Protein Kinase B; LD, Lipid droplet; PKA, Protein kinase A; p62, Sequestosome 1; LC3B, Microtubule-Associated Protein 1 Light Chain 3B; ULK1, UNC-51-like kinase 1; ER stress, Endoplasmic Reticulum stress; NASH, Non-alcoholic Steatohepatitis; NAFL, Non-alcoholic Fatty Liver; HCC, Hepatocellular Carcinoma; HFD, high-fat diets.

In summary, maintaining a healthy lifestyle with regular eating habits and moderate exercise may effectively prevent liver cancer. Lipophagy plays a central role in these processes by regulating lipid metabolism and maintaining hepatic health (as shown in Figure 2).

## 5 Conclusion

HCC, as the terminal stage of chronic liver disease, arises from multifactorial pathogenesis including viral hepatitis (e.g., HBV), metabolic dysfunction (e.g., NAFLD), and environmental exposures (Liao et al., 2017). While preventive strategies like HBV vaccination mitigate risk, they cannot fully eliminate HCC development, necessitating novel therapeutic approaches (Lou et al., 2017).

Lipophagy serves as a critical regulator with dual-stage functions in tumorigenesis, suppressing early tumor initiation through cytotoxic lipid clearance and chemosensitization while driving advanced progression via FFA-mediated metabolic reprogramming. Key mediators include transcriptional regulators like TFEB and C/EBPβ alongside the PI3K/AKT/mTOR signaling axis. While preclinical data support the potential of lipophagy modulators such as metformin, quercetin, and PLIN2 inhibitors, clinical application remains hindered by HCC heterogeneity, stage-dependent efficacy variations, and drug delivery constraints. Research priorities should focus on characterizing stage-specific molecular profiles (e.g., CD36-high versus TFEB-amplified subtypes), designing context-responsive therapeutics, establishing robust biomarkers (LC3-II), and optimizing targeted delivery platforms. These stage-selective mechanisms represent a viable therapeutic strategy for metabolic syndrome-associated HCC, addressing a pressing clinical challenge.
