# Supplementary material for: The role of lipophagy in liver cancer: mechanisms and targeted therapeutic interventions
Source: Front Cell Dev Biol. 2025 Jul 8;13:1562542. doi: 10.3389/fcell.2025.1562542 (PMC12279770; doi:10.3389/fcell.2025.1562542)
Supplement: Supplementary file 1 [file Table1.docx]

Supplementary Material

# Supplementary Figures and Tables

## Supplementary Tables

**Supplementary Table 1**. **Overview of Core Molecules and Signaling Pathways in Lipophagy Regulation**

| **Key Molecules and Signaling Pathways in Lipophagy Regulation** | **Introduction** |  | **Mechanism of Action in Lipophagy** | **Outcome of Action** |
| --- | --- | --- | --- | --- |
| PNPLA Family | Non-secretory proteins of the patatin-like phospholipase family |  | Initiate Lipophagy | Lipid droplet degradation, energy supply |
| ATGL | Adipose Triglyceride Lipase |  | Regulate lipid droplet breakdown through SIRT1 activity to promote lipophagy | Promote lipid droplet breakdown, energy supply |
| RabGTPases | Rab, also known as targeting GTPase, belongs to the monomeric GTPase family |  | Regulating the transport and fusion of membrane vesicles in the process of lipophagy | Promote the process of lipophagy |
| TFEB | E-box transcription factor |  | Transcriptional regulation of lipophagy-related gene expression | Promote lysosome formation and lipophagy-related gene expression |
| TFE3 | Transcription factor binding to IGHM enhancer 3 |  | Transcriptional regulation of lipophagy-related gene expression | Promote the expression of lipophagy-related genes |
| FOXOs | Forkhead box O transcription factors |  | Transcriptional regulation of lipophagy-related gene expression | Promote the expression of lipophagy-related genes |
| LAMP2 | Lysosome-associated membrane protein 2 |  | Affect the formation of autolysosomes | Enlarge autolysosomes and enhance lipophagy |
| Oleic acid (OA) | Oleic acid |  | Inhibit the colocalization of LC3 and LAMP1, preventing the formation of autolysosomes | Reduce lipid accumulation, restore autophagic flux |
| SREBP-3 | Sterol regulatory element-binding protein 3 |  | Mediated PNPLA8-driven lipophagy | Promote lipophagy |
| LC3 | Microtubule-associated protein 1 light chain 3 |  | Interacts with ATGL, promotes the movement of ATGL to LD, induces lipophagy | Promote lipophagy |
| p62 | Ubiquitin-binding protein; chelator 1, also known as SQSTM1 |  | p62 undergoes self-polymerization and recruits LC3^+^ phagocytes to the lipophagy site | Leads to lysosomal degradation |
| AMPK | AMP-activated protein kinase |  | When activated, promotes lipophagy and inhibits mTOR | Promotes lipophagy |
| mTOR | Mammalian target of rapamycin |  | Inhibition of mTOR is a trigger condition for lipophagy | Other pathways (such as AMPK) can promote lipophagy |
| LDs | Lipid droplets |  | Substrates of lipophagy are degraded to release free cholesterol and fatty acids | Lipid degradation, energy supply |
| PPARα | Peroxisome proliferator-activated receptor α |  | Activate lipid metabolism and lipophagy | Promote lipid metabolism and autophagy |
| ULK1 | UNC-51-like kinase 1 |  | Form the ULK1 complex to activate lipophagy | Activate lipophagy |

**Supplementary Table 2. Effects and Results of Substances Related to Drug Resistance and Lipophagy**

| **Substances Affecting Drug Resistance** | **Impact on Lipophagy** | **Resulting Outcome** |
| --- | --- | --- |
| CD36 | Promote lipophagy | Expression of Chemotherapy Resistance Signals |
| Sorafenib | Inhibition of Lipophagy | Reduction of Drug Resistance |
| PLIN3 | PLIN3 Depletion, Impairment of Lipophagy | Leads to Drug Resistance |
| Aurora Kinase A (AURKA) | Activation of Lipophagy | Induction of Drug Resistance |
| MicroRNA (miR)-425 | Promote lipophagy | Induction of Drug Resistance |

**Supplementary Table 3. Overview of the impact of drugs on the mechanism and outcomes of lipophagy**

| **Drugs** | **Mechanism of action** | **Effects on lipophagy** |
| --- | --- | --- |
| **Western medicine** | | |
| Metformin | Induces activation of AMPK; AMPK-SIRT1 axis; disrupts mRNA stability | Promote |
| Geniposide | By inhibiting the PARP1/PI3K/AKT signaling pathway | Promote |
| Olaparib | A PARP1 inhibitor, inhibits PARP1 | Promote |
| Sodium palmitate | Inhibit the expression of SCD1 (a key enzyme controlling lipid metabolism) in hepatocytes | Promote |
| Atorvastatin | By upregulating AMPK phosphorylation and downregulating the phosphorylation of mammalian targets of rapamycin | Promote |
| Caffeine | Inhibition of PI3-AKT, in turn, through activation of the ULK1 complex | Promote |
| PPARα Agonist Fenofibrate | Activate the calcineurin and CaMKKβ-AMPK-ULK1 pathway, promoting the dephosphorylation and nuclear translocation of TFEB and TFE3 | Promote |
| Glycolytic inhibitor PFK158 | Targeting glycolysis and lipogenesis pathways | Promote |
| p62Agonist | Through the p62-mediated N-degron pathway | Promote |
| SCD1 inhibitor CAY10566 | Enhanced AMPK activity | Promote |
| Sea cucumber plasmalogen | Alleviates lipid accumulation, stimulates dynamic remodeling of the autophagosome membrane, with a significant decrease in LC3 II/I ratio and p62 levels | Promote |
| Abrus agglutinin (AGG) | Upregulation of SIRT1 leads to deacetylation of Lys residues on the cytoplasmic domain of LAMP1 | Promote |
| Docosahexaenoic acid (DHA) | The combination of induced LDs regeneration and inhibition of lipophagy signaling increases the production of autophagosomes and autophagic flux | Inhibit |
| Bufalin rod-shaped mesoporous silica nanoparticles(BA-rMSNs) | Inhibits cell proliferation and induces cell death, enhancing tumor targeting | Inhibit |
| Sulfated glucuronomannanhexamer G6S1 | Enhance activation of PPARα expression | Promote |
| Sirt1 Agonist SRT1720 | Promote Sirt1 expression, accompanied by overexpression of Foxo1 and Rab7 | Promote |
| Rab7 Agonist ML-098 | Promote Rab7 expression | Promote |
| Alpelisib and Digoxin | Digoxin as an ATP-dependent Na^+^-K^+^ transporter inhibitor; Alpelisib as a PI3K inhibitor | Promote |
| **Traditional Chinese medicine** | | |
| Naringin | Restoration of TFEB-mediated lysosomal biogenesis | Promote |
| Gualou-Xiebai (GLXB) | By inhibiting P2RY12 activation | Promote |
| Zexie Decodion | Regulate the Akt/TFEB signaling pathway | Promote |
| Formononetin | Activate AMPK and promote subsequent nuclear translocation of Transcription Factor EB (TFEB) | Promote |
| Dihydromyricetin (DHM) | Increase the interaction and co-localization of p62/SQSTM-1, LC3B, and PLIN1 proteins | Promote |
| Ginsenoside Compound K (CK) | Activate AMPK/ULK1 signaling pathway | Promote |
| Ajugol | Enhance TFEB-mediated lysosomal biogenesis | Promote |
| Tetrahydrocurcumin (THC) | Induce TFEB nuclear translocation by upregulating lysosomal biogenesis through inhibition of mTORC1 | Promote |
| Phillygenin (PHI) | Regulate the Ca^2+^ -calcineurin-TFEB axis in hepatocytes | Promote |
| Quercetin | Activate AMPK signaling, reduce PLIN2 levels, induce AMPK activity, and increase LC3II and PLIN2 proteins | Promote |
| Polydatin | Inhibits mTOR signaling and upregulates the expression and activity of TFEB | Promote |
| Astragaloside I (ASI) | Inhibits the protein level of p-TFEB (ser211) expression and promotes TFEB nuclear translocation. | Promote |
| Nobiletin (NOB) | Through TFEB-mediated lysosomal biogenesis | Promote |
| Honokiol | Activates the SIRT3-AMPK-lipophagy axis | Promote |
| Sulforaphane | Activates the AMPK-mTOR-ULK1 pathway signaling in adipocytes | Promote |
| Baicalin and N-acetylcysteine (NAC) | Inhibition of mROS-mediated CMA levels through mitochondrial transcription factor A (TFAM)-choline | Promote |
| Stevia and Stevioside | Increased levels of PPARα | Promote |
| Valeriana fauriei and its iridoids | By inhibiting mTORC1 activity | Promote |
